# Identification and validation of IRF6 related to ovarian cancer and biological function and prognostic value

**DOI:** 10.1186/s13048-024-01386-4

**Published:** 2024-03-16

**Authors:** Shihao Hong, Ni Fu, Shanliang Sang, Xudong Ma, Fangying Sun, Xiao Zhang

**Affiliations:** 1 Department of Obstetrics and Gynecology, Sir Run Run Shaw Hospital, School of Medicine, Zhejiang University, Hangzhou, 310016 China; 2 Key Laboratory of Reproductive Dysfunction Management of Zhejiang Province, Hangzhou, 310016 China; 3 Zhejiang Province Clinical Research Center for Obstetrics and Gynecology, Hangzhou, 310016 China; 4 Department of Obstetrics and Gynecology, Huangyan Hospital of Chinese Medicine, Taizhou, Zhejiang Province 318020 China

**Keywords:** Ovarian cancer, IRF6, Prognosis marker, WCGNA, Bioinformatics

## Abstract

**Background:**

Ovarian cancer (OC) is a severe gynecological malignancy with significant diagnostic and therapeutic challenges. The discovery of reliable cancer biomarkers can be used to adjust diagnosis and improve patient care. However, serous OC lacks effective biomarkers. We aimed to identify novel biomarkers for OC and their pathogenic causes.

**Methods:**

The present study used the differentially expressed genes (DEGs) obtained from the “Limma” package and WGCNA modules for intersection analysis to obtain DEGs in OC. Three hub genes were identified—claudin 3 (CLDN3), interferon regulatory factor 6 (IRF6), and prostasin (PRSS8)—by searching for hub genes through the PPI network and verifying them in GSE14407, GSE18520, GSE66957, and TCGA + GTEx databases. The correlation between IRF6 and the prognosis of OC patients was further confirmed in Kaplan-Miller Plotter. RT-qPCR and IHC confirmed the RNA and protein levels of IRF6 in the OC samples. The effect of IRF6 on OC was explored using transwell invasion and scratch wound assays. Finally, we constructed a ceRNA network of hub genes and used bioinformatics tools to predict drug sensitivity.

**Results:**

The joint analysis results of TCGA, GTEx, and GEO databases indicated that IRF6 RNA and protein levels were significantly upregulated in serous OC and were associated with OS and PFS. Cell function experiments revealed that IRF6 knockdown inhibited SKOV3 cell proliferation, migration and invasion.

**Conclusion:**

IRF6 is closely correlated with OC development and progression and could be considered a novel biomarker and therapeutic target for OC patients.

**Supplementary Information:**

The online version contains supplementary material available at 10.1186/s13048-024-01386-4.

## Introduction

Ovarian cancer (OC), one of the most common gynecological cancers, ranks seventh worldwide among malignant tumors and fifth among female cancer-related deaths [1, 2]. According to the World Health Organization, over 220,000 people worldwide are diagnosed with OC annually, and 150,000 die from OC [3]. Ultrasound is the most widely used method for detecting early-stage OC [4]. Ultrasound assessment of tumor echo-morphology (cyst wall thickness, papillary projections, septae, solid/cystic components within cysts, bilateralism, cyst diameter, etc.) [5]. Several methods, such as surgery, chemotherapy, radiotherapy, and molecular targeted therapy, treat OC [6]. Although the results of OC treatment have improved with the diversification of treatment strategies, most OC patients are diagnosed at a late stage with cancer metastasis, leading to a very low 5-year overall survival (OS) rate or recurrence [7]. Therefore, early detection and diagnosis are crucial for the treatment outcomes of OC patients. Although classic targets, such as PD1 and EGFR, have been proven to be available for treating OC patients, not all patients are sensitive to these targeted therapies [8]. Consequently, to better diagnose and treat OC patients, we must explore new biomarkers to guide healthcare professionals in predicting patient prognosis and developing precise, personalized treatment plans, which may contribute to an increase in the survival rate.

Recently, the establishment of large databases such as TCGA, GTEx, and GEO has enabled researchers to better analyze information in the tumor genome, such as mutated genes, fusion genes, CNV, and epigenetic changes, to screen for meaningful prognostic biomarkers [9, 10]. Therefore, developing bioinformatics has aided researchers in deepening their understanding of tumor occurrence and development and in conducting pathological mechanism research on diseases. Conventional differential expression analysis based on the “Limma” package can only identify the changes in individual genes within a disease. However, cancer is a multidimensional and complex process that involves multiple genes. This gap may be filled by Weighted Gene Co-expression Network Analysis (WGCNA). WGCNA is widely used to analyze transcriptome data. It clusters genes into different modules based on the correlations among differentially expressed genes and establishes connections between genes and diseases [11, 12]. WCGNA can also be used to analyze the relationship between genes and clinical features of diseases [9, 10].

In this study, we obtained OC-related microarray datasets from the GEO database. Through collaborative analysis of the “Limma” package and WCGNA, we obtained differentially expressed genes (DEGs) between normal ovarian and OC tissues. We then conducted functional enrichment analysis of DEGs through databases such as GO, KEGG, Reactome, and WikiPathways to deepen our understanding of the pathological mechanisms of OC. Next, hub genes were identified using the STRING database and Cytoscape. We then validated the hub genes using external GEO and TCGA + GTEx datasets and found that only CLDN3, IRF6, and PRSS8 had consistent trends in all datasets. Furthermore, the Kaplan–Meier Plotter database revealed that CLDN3 and IRF6 were associated with OS and PFS in OC patients. We conducted a series of analyses, including developing a competitive endogenous RNA (ceRNA) regulatory network, immune-related analysis, and drug sensitivity analysis. Finally, we confirmed the role of IRF6 in the oncogenic effects of OC. In conclusion, we discovered that IRF6 could serve as a biological marker for OC, which is could provide theoretical and experimental evidence for future clinical research in OC.

## Methods

### Gene expression data collection

The GEO database (https://www.ncbi.nlm.nih.gov/geo/) was used to analyze mRNA expression levels in OC patients. This study used the datasets GSE66957 (Normal: 12, Tumor:57), GSE14407 (Normal: 12, Tumor: 12), and GSE18520 (Normal: 10, Tumor: 53). The TCGA database (https://portal.gdc.cancer.gov/) only contains cancer tissue samples from 587 OC patients; therefore, we introduced 88 normal ovarian tissue samples from the GTEx database (https://www.gtexportal.org/) as a control for TCGA-OC. All detailed information of patients were listed in Supplementary File 1.

### Identification of differentially expressed genes with Limma

Differential gene analysis was performed on GSE66957 using the “Limma” package, with |logFC|> 1 and a *p*-value < 0.05 indicating significance. More details are in Supplementary File 2.

### Weighted Gene Co-Expression Network Analysis (WGCNA)

This study analyzed GSE66957 using WGCNA and constructed a co-expressed gene module. The soft threshold of GSE66957 was set to 5, the minimum module gene was set to 30, the module merge threshold was set to 0.25, and MM values greater than 0.8 were considered disease-characteristic genes. More details are in Supplementary File 3. ‘Venny’ (https://bioinfogp.cnb.csic.es/tools/venny/) was used to create the Venn diagram. Common DEGs were used for subsequent analyses. More details are in Supplementary File 4.

### Functional analyses

Following the identification of common DEGs, we conducted a functional enrichment analysis. The following databases were used: GO, KEGG, Reactome, and WikiPathways. These databases were used to determine the biological pathways and functions of common DEGs. Statistical significance was set at *p* < 0.05. More details are in Supplementary File 5.

### PPI network construction

A PPI network was developed using the STRING database (https://string-db.org) to analyze the interactions among DEGs. If the confidence setting was greater than 0.7, non-hidden proteins were hidden.

### Screening of hub genes

The results generated using the string database were imported into Cytoscape for analysis, and the plugin cytoHubba was used to analyze hub genes. The top 10% of genes calculated through MCC and Degree algorithms were considered hub genes. More details are in Supplementary File 6.

### Verification of hub genes

GSE14407 and GSE18520 from the GEO database were obtained to further analyze hub gene expression. The GEPIA2.0 database (GEPIA2.cancer-pku.cn) integrates sequencing data from TCGA and CTEx databases. The GEPIA2.0 database was used to evaluate the expression levels of hub genes.

### Prognostic signature of hub genes

The Kaplan–Meier plotter (www.kmplot.com) was used to perform OS, progression‐free survival (PFS), and post-progression survival (PPS) analyses of hub genes.

### Immune analysis

The TIMER algorithm was performed to calculate the abundances of B cells, Macrophage cells, Dendritic cells, Neutrophil cells, CD4^+^ T cells, and CD8^+^ T cells. The MCP Counter algorithm was used to calculate the abundance of ten immune infiltrating cells. The Stromal, ESTIMATE, and immune scores were calculated to estimate the immune status in OC using the ESTIMATE algorithm. Furthermore, CIBERSORT was used to distinguish between 22 human immune cell phenotypes.

### GeneMANIA analysis

GeneMANIA (http://www.genemania.org) is a protein database that analyzes the core proteins and their interactions. This database explains the functional networks between genes and promotes research on their functions.

### Construction of ceRNA network

The Starbase (https://starbase.sysu.edu.cn/starbase2/index.php) and TargetScan (http://www.targetscan.org/vert_72/) databases were used to predict the hub gene miRNAs. Common miRNAs were used in the Starbase and TargetScan databases to predict lncRNAs. The collected miRNAs, lncRNAs, and hub genes were connected to construct a ceRNA regulatory network. More details are in Supplementary File 7.

### Drug sensitibity analysis

The molecular and pharmacological data of the NCI-60 cancer cell line were downloaded from CellMiner (https://discover.nci.nih.gov/cellminer/home.do), and the correlation was evaluated between IRF6 mRNA expression levels and drug sensitivity using Pearson’s correlation coefficient.More details are in Supplementary File 8.

### Ovarian cancer tissue and immunohistochemistry

Tumor and normal samples from ovarian cancer patients were obtained from the Affiliated Sir Run Run Shaw Hospital of Zhejiang University School of Medicine, all detailed information of patients are listed in Supplementary File 9. Paraffin-embedded tissues from Tumor and normal samples were sectioned for immunohistochemistry. Briefly, sections were deparaffinized in xylene and dehydrated using a graded ethanol series. After deparaffinization and rehydration, the endogenous peroxidase activity was blocked with 0.3% hydrogen peroxide in methanol for 30 min. Following heat-mediated antigen retrieval in sodium citrate buffer (pH 6.5), the blocked sections were incubated with anti-IRF6 antibody (1:100 dilution; Santa Cruz Biotechnology, Santa Cruz, CA) overnight at 4 °C. The signal was amplified by adding 3–3'-diaminobenzidine to the avidin–biotin-peroxidase complex (Beyotime, China). Finally, the sections were counterstained with hematoxylin and observed under a microscope.

### Cell culture and transfection

The human OC cell line SKOV3 (Homo sapiens, human; RRID: CVCL_0532) was purchased from the Cell Bank of the Chinese Academy of Sciences (Shanghai, China). The cell line was authenticated by STR profiling. The cells were cultured in DMEM supplemented with 10% FBS, 100 U/mL of penicillin sodium, and 100 mg/mL of streptomycin sulfate. The cells were then incubated at 37 °C with 5% CO_2_. SKOV3 cells were cultured in 12-well plates and transfected with si-NC or si-IRF6 using Lipofectamine 3000 (Invitrogen, Carlsbad, CA, USA), according to the manufacturer’s instructions. All experiments were performed using mycoplasma-free cells. The sequences of siRNA-IRF6 and si-control were 5ʹ- CTGAGCATATTACCAATGA -3ʹ and 5ʹ- TTCTCCGAACGTGTCACGT -3ʹ.

### Transwell invasion and scratch assays

The migration and invasion of the human OC cell line SKOV3 were evaluated using a Transwell assay with a 24-well, 8-µm pore size Transwell plate (Costa, Cambridge, MA). Before seeding the cells onto the membrane for invasive testing, 100 μL of 1:10 DMEM filtered Matrix (BD, USA) was added to each well and incubated at 37 °C for 6 h, then SKOV3 cells transfected with si-NC or si-IRF6 (1 × 10^5^ cell/well) were inoculated in the upper chamber (serum-free medium), and 20% FBS culture medium was added to the lower chamber. After 24 h, fixation, and crystal violet staining were performed, and the migrated cells were photographed under a microscope. To assess the cell migration properties of SKOV3, SKOV3 cells transfected with si-NC or si-IRF6 (1 × 10^5^ cell/well) were grown until confluence. When the cell fusion reached 80%, the P200 pipette tip was used perpendicular to the horizontal line to scratch, and the scratch assay was observed under an inverted microscope at 0, 24, and 48 h, respectively. To determine cell migration ability, one examined the wound healing percentage (distance migrated/original wound distance × 100%).

### Cell proliferation assay based on CCK8 (Cell Counting Kit-8) assay

The human OC cell line SKOV3 were seeded in 96-well plats with 2 × 10^4^ cell/well and were cultured for 0 h, 24 h, 48 h or 72 h. 10 μL of CCK8 solution (Yeasen, Shanghai, China) were added to each well and incubated for 2 h at 37 °C. A microplate reader was used (Thermo Fisher Scientific, Waltham, USA) to measure optical density at 450 nm (OD450nm).

### RT-qPCR

Human ovarian cancer tissues and adjacent noncancerous tissues were collected, and total RNA was extracted using TRIzol Reagent (Invitrogen) according to the kit instructions. 1 μg of total RNA was reverse‐transcribed to cDNA using the cDNA Reverse Transcription kit (Vazyme, Nanjing, China) at 37 °C for 60 min. Real-time PCR was performed using TB Green™ Premix Ex Taq™ II (RR420A; Takara, China) in a Roche LightCycler®96 qRT-PCR system according to the manufacturer’s protocol. Primers for IRF6 (interferon regulatory factor 6) were, forward: 5′- CCCCAGGCACCTATACAGC-3′ and reverse: 5′- TCCTTCCCACGGTACTGAAAC-3′; GAPDH was used as an internal reference for the calculation of IRF6 RNA expression, expression difference was calculated using 2^−ΔΔCT^ method.

### Statistical analysis

GraphPad Prism 8.0 software was used for data analysis. All data are expressed as mean ± standard deviation (SD) and mean ± standard error of the mean (SEM). Group differences with *P* < 0.05 were considered statistically significant using Student's t‐test.

## Results

### Common DEG Identification in OC

**Fig. 1:**
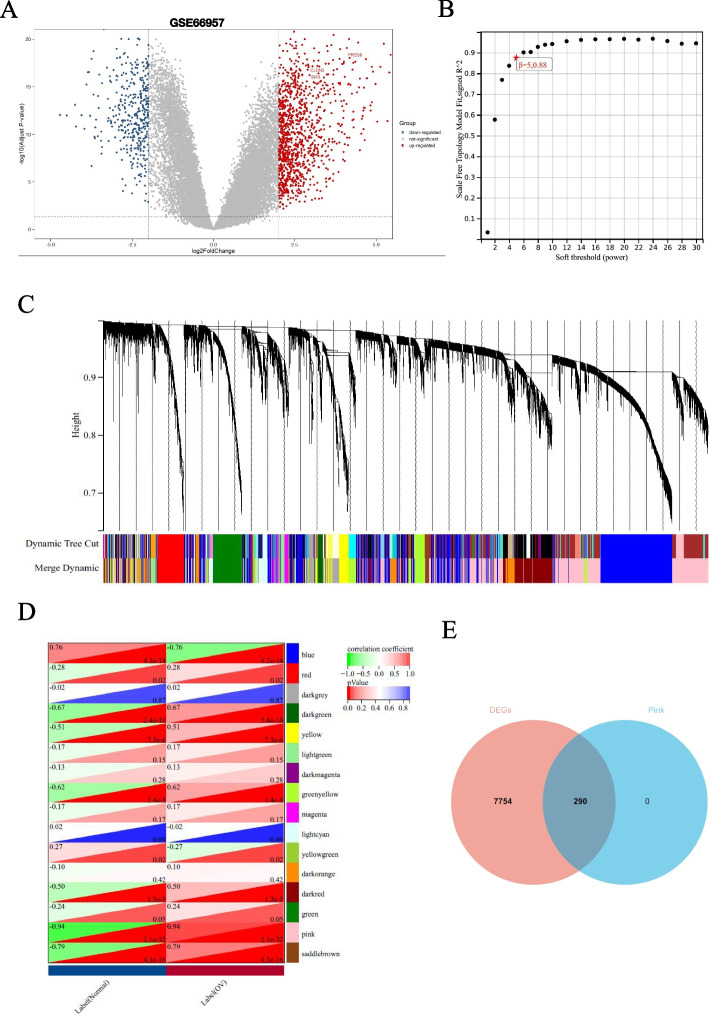
WGCNA and DEGs analysis. **A** DEGs between the Normal and OC groups are shown on a volcano from GSE66957 data series downloaded from GEO database. **B** Scale-free fit index as a function of soft threshold power. **C** Hierarchical clustering of OC genes that are expressed similarly. **D** Module–trait relationships. **E** Venn diagram shows common genes from DEGs and significant module of WGCNA

The “Limma” package and WGCNA were jointly used to search for differentially expressed genes in OC. According to the analysis results of the “Limma” package, there were 8045 differentially expressed genes in GSE66957, of which 5273 DEGs are upregulated, and 2771 DEGs are downregulated (Fig. 1A). By setting a soft threshold of 5 (Fig. 1B) and using WGCNA, we identified 16 modules, each with similar gene co-expression characteristics of genes (Fig. 1C). The Spearman correlation coefficient between the modules and features was used to calculate the importance of the modules (Fig. 1D). The genes in the pin module with the most significant *P*-values were selected and intersected with the 8045 genes aforementioned to obtain common DEGs for subsequent analysis (Fig. 1E).

### Functional analysis of common DEGs

**Fig. 2:**
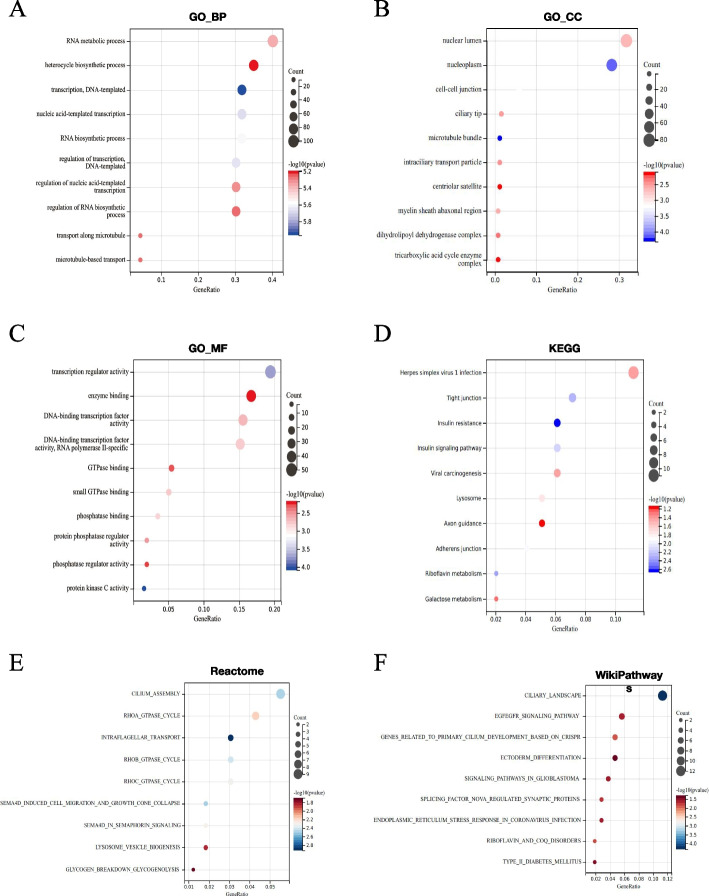
Functional analysis. **A** GO terms (Biological Process, BP). **B** GO terms (Cellular Component, CC). **C** GO terms (Molecular Function, MF). **D** KEGG Enrichment. **E** Reactome Enrichment. **F** WikiPathways Enrichment

To further understand the pathological mechanism of OC, we conducted an enrichment analysis of the biological functions and signaling pathways involved in common DEGs in OC. The results of GO analysis indicated that the following biological processes were influenced by common DEGs: translation, DNA template, nuclear acid template translation, regulation of translation, DNA template, RNA biosynthetic process, RNA metabolic process, regulation of nuclear acid template transfer, transport along the microtubule, microtubule-based transport, regulation of RNA biosynthetic process, and heterocycle biosynthetic process (Fig. 2A). Furthermore, we used the GO database to analyze cellular components (Fig. 2B) and molecular functions involved in common DEGs (Fig. 2C). KEGG analysis revealed that these common DEGs were associated with insulin resistance, riboflavin metabolism, tight junctions, insulin signaling pathways, adherens junctions, lysosomes, viral carcinogenesis, herpes simplex virus 1 infection, galactose metabolism, and axon guidance signaling pathways (Fig. 2D). We also conducted a signal pathway enrichment analysis in the Reactome (Fig. 2E) and WikiPathways (Fig. 2F). Based on the aforementioned functional enrichment analysis results, these common DEGs were related to transcriptional regulation, microtubules, and metabolism.

### Identification of Hub Genes

**Fig. 3:**
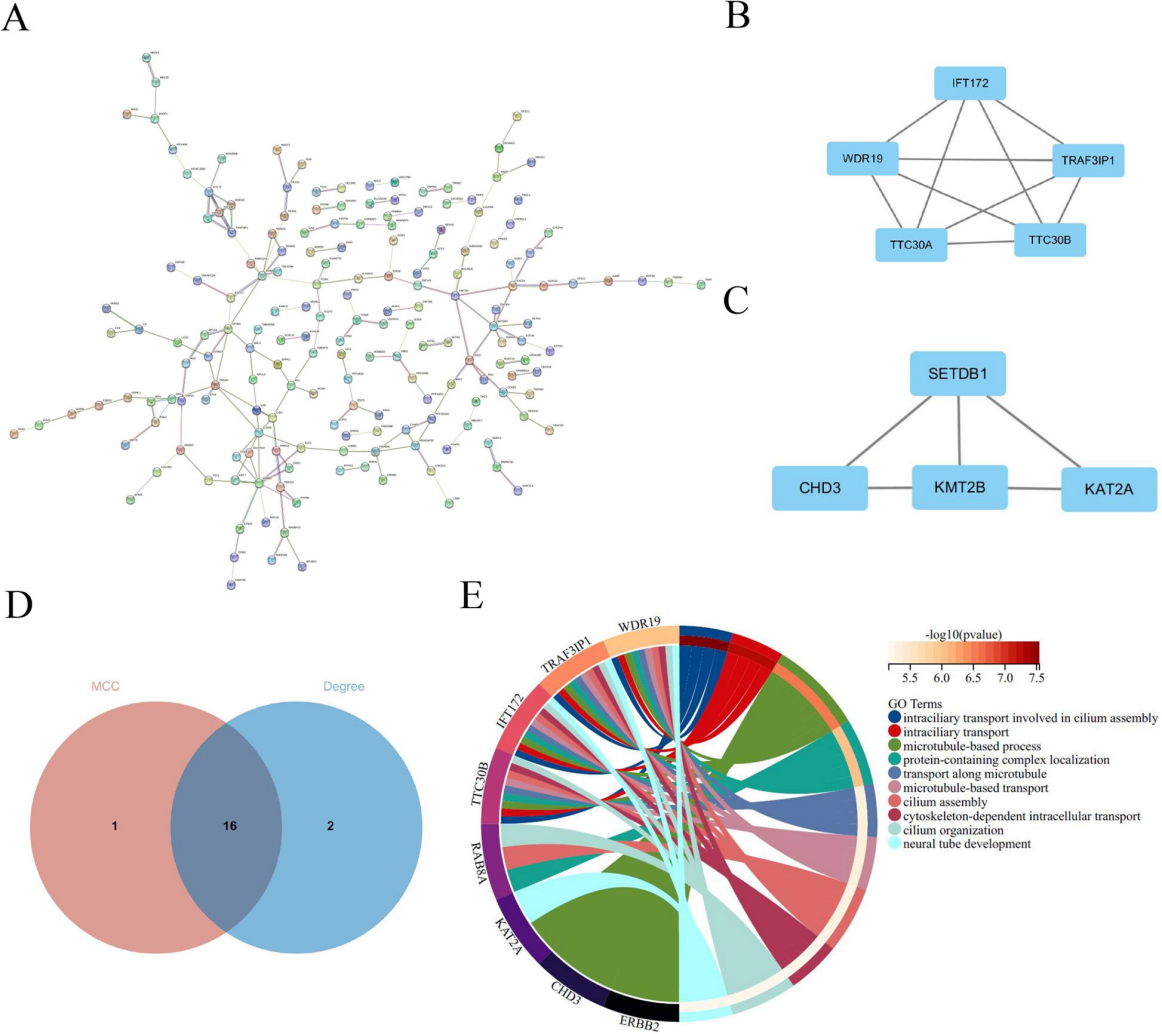
CytoHubba analysis of PPI network. **A** 290 DEGs were filtered into the DEGs PPI network through STRING database. **B** Identify core network-1 through MCODE. **C** Identify core network-2 through MCODE. **D** Identifying hub genes through MCC and Degree algorithms. **E** Functional analysis of common hub genes

Using the STRING database, 290 common DEGs were used to construct the PPI network (Fig. 3A). The generated file was imported into Cytoscface, an MCODE plugin, for analyzing the core protein network (Figs. 3B-C). MCC and Degree algorithms were used to identify the top 10% of genes using the hub gene analysis method derived from the cytoHubba plugin (Fig. 3D). After intersecting these genes, we identified 16 hub genes. This finding suggests that these 16 genes are essential for OC. On the other hand, we conducted KEGG analysis on these 16 genes to further explore the pathological mechanisms in OC.

**Fig. 4:**
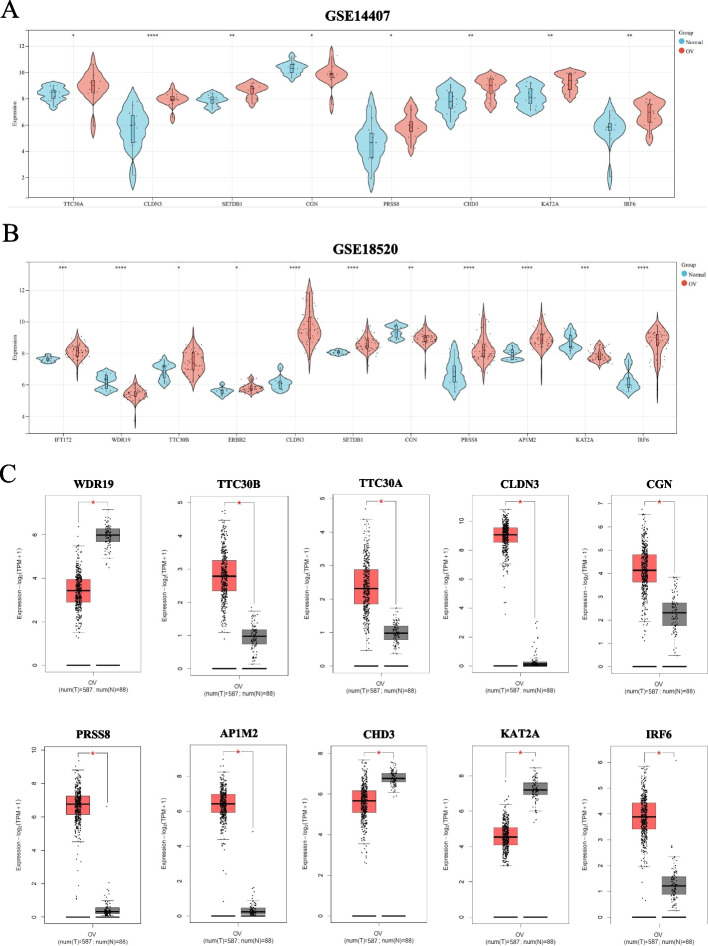
The identification of hub genes. The expressions of common hub genes in (**A**) GSE14407, (**B**) GSE18520, (**C**) TCGA + GTEx. The GSE14407 and GSE18520 data series downloaded from GEO database. (* means *P* < 0.05; **means *P* < 0.01; *** means *P* < 0.001; **** means *P* < 0.0001. Student’s t-test, Error bars are ± SEM)

Next, we selected GSE14407 and GSE18520 datasets from the GEO database to confirm their differential expression (Figs. 4A-B). Meanwhile, considering that there were only OC samples in the TCGA database, we introduced ovarian samples from the GTEx database as controls and calculated their differential expression in GEPIA2.0 (Fig. 4C). We only presented differentially expressed genes. After analyzing the three datasets, we believe that the genes CLDN3, IRF6, and PRSS8, with common expression trends, play an essential role in OC pathogenesis.

**Fig. 5:**
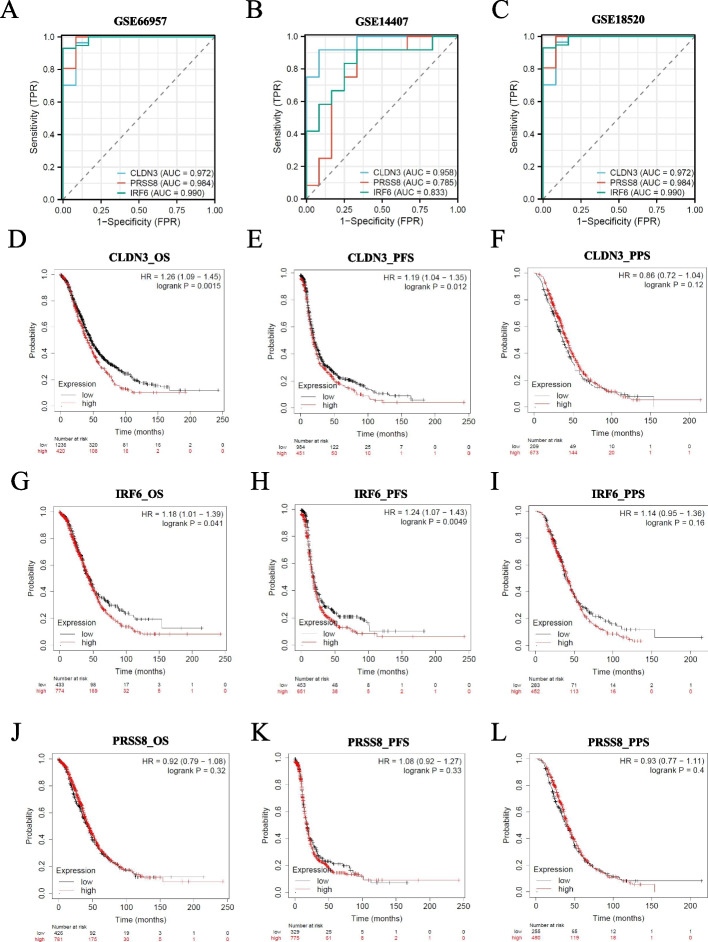
The Survival analysis of CLDN3, IRF6 and PRSS8. The receiver operating characteristic curve analysis of hub genes in (**A**) GSE66957, (**B**) GSE14407, (**C**) GSE18520. The GSE66957, GSE14407 and GSE18520 data series downloaded from GEO database. The survival analysis in KM plotter: (**D**) OS of CLDN3. **E** PFS of CLDN3. **F** PPS of CLDN3. **G** OS of IRF6. **H** PFS of IRF6. **I** PPS of IRF6. **J** OS of PRSS8. **K** PFS of PRSS8. **L** PPS of PRSS8.

We conducted ROC diagnostic analysis of CLDN3, IRF6, and PRSS8 in GSE66957, GSE14407, and GSE18520 datasets. The results displayed that the area under the curve for these three genes was greater than 0.78, indicating that these three genes have excellent diagnostic value. Among them, CLDN3 and IRF6 performed better than PRSS8 (Figs. 5A-C). Finally, we investigated the relationship between CLDN3, IRF6, and PRSS8 expression levels and patient prognosis. The findings of the Kaplan–Meier plotter indicate that the differences in expression levels of CLDN3 (Figs. 5D-F) and IRF6 (Figs. 5G-I) are related to OS and PFS in OC patients. The prognostic value of PRSS8 was not significant (Figs. 5J-L).

### Immune cell infiltration in OC

**Fig. 6:**
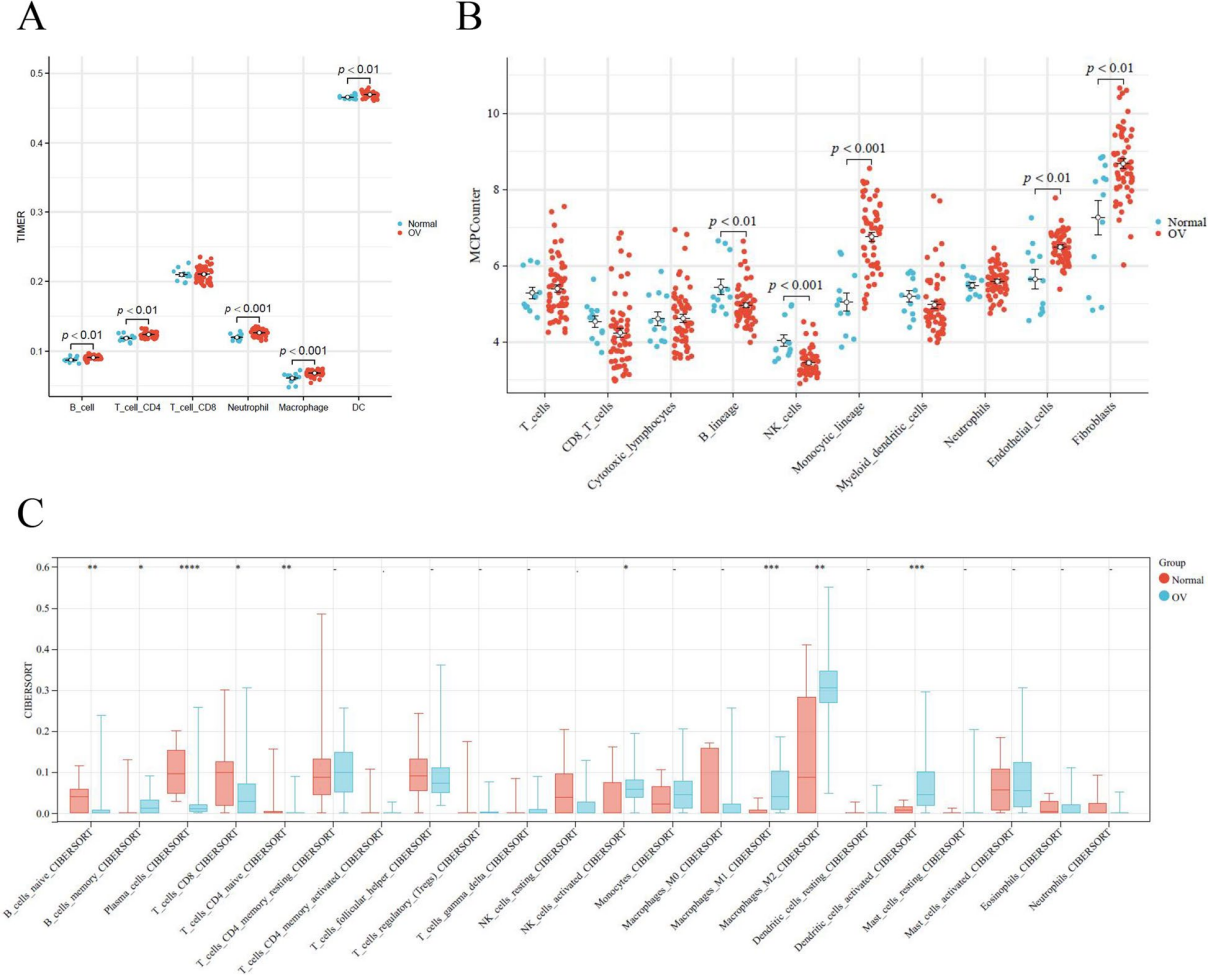
Immune cell infiltration analysis. **A** Sabundance of six immune filtrating cells evaluated by TIMER. **B** Iimmune filtrating cells evaluated by MCPCounter. **C** 22 distinct immune cell subtype compositions in OC and Normal samples

Because the immune system plays an essential role in the pathogenesis of OC, we observed changes in the immune microenvironment of patients with OC by evaluating the level of infiltration of immune cells. First, using the TIMER algorithm, we found that in OC patients, there were differences in various immune cells except for CD8 T cells (Fig. 6A). This finding indicates that the immune system in OC patients indeed exhibits abnormalities. Subsequently, we used the MCP Counter algorithm to continue analyzing the level of immune cell infiltration and found that CD8_ T_ The cells did not exhibit any difference (Fig. 6B). To comprehensively analyze the level of immune cell infiltration, we used the CIBERSORT algorithm to evaluate the differences in 22 types of immune cells between normal individuals and OC patients. The results demonstrated that the proportion of B cells in OC patients decreased while the proportion of macrophages and DC cells increased (Fig. 6C). These findings demonstrate the importance of immunology in OC development.

### GeneMANIA and ceRNA network for CLDN3, IRF6 and PRSS8

**Fig. 7:**
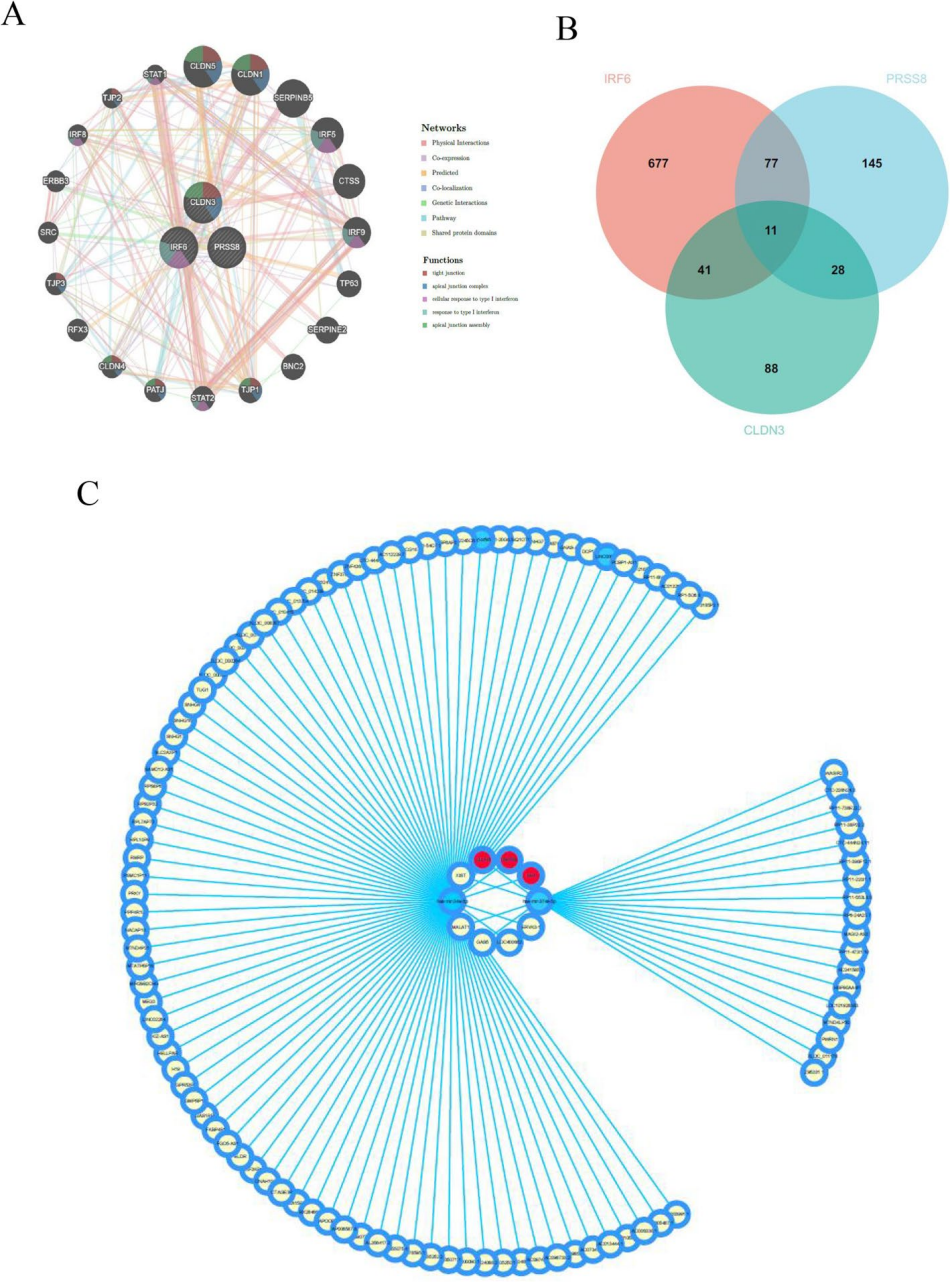
Construction of ceRNA network. **A** The protein–protein interaction network of CLDN3, IRF6 and PSSR8 by GeneMANIA Analysis. **B** The Venn diagram depicts the overlap between CLDN3, IRF6 and PSSR8. **C** The integrated lncRNA-miRNA-hub genes network

Co-expression network and biological processes of CLDN3, IRF6, and PRRS8. GeneMANIA indicates that these mechanisms are closely related to the cell junctions. Gene expression is jointly regulated by miRNAs and lncRNA. CeRNA is a novel regulatory mechanism (Fig. 7A). The miRNAs of the three hub genes were predicted using TargetScan and Starbase, and the intersecting miRNAs were used for subsequent lncRNA prediction (Fig. 7B). A ceRNA network was constructed using CLDN3, IRF6, PRSS8, two miRNAs (miR-34a-5p and miR-374a-5p), and 109 lncRNAs (Fig. 7C).

### Validation of IRF6 in histological and cellular experiments

**Fig. 8:**
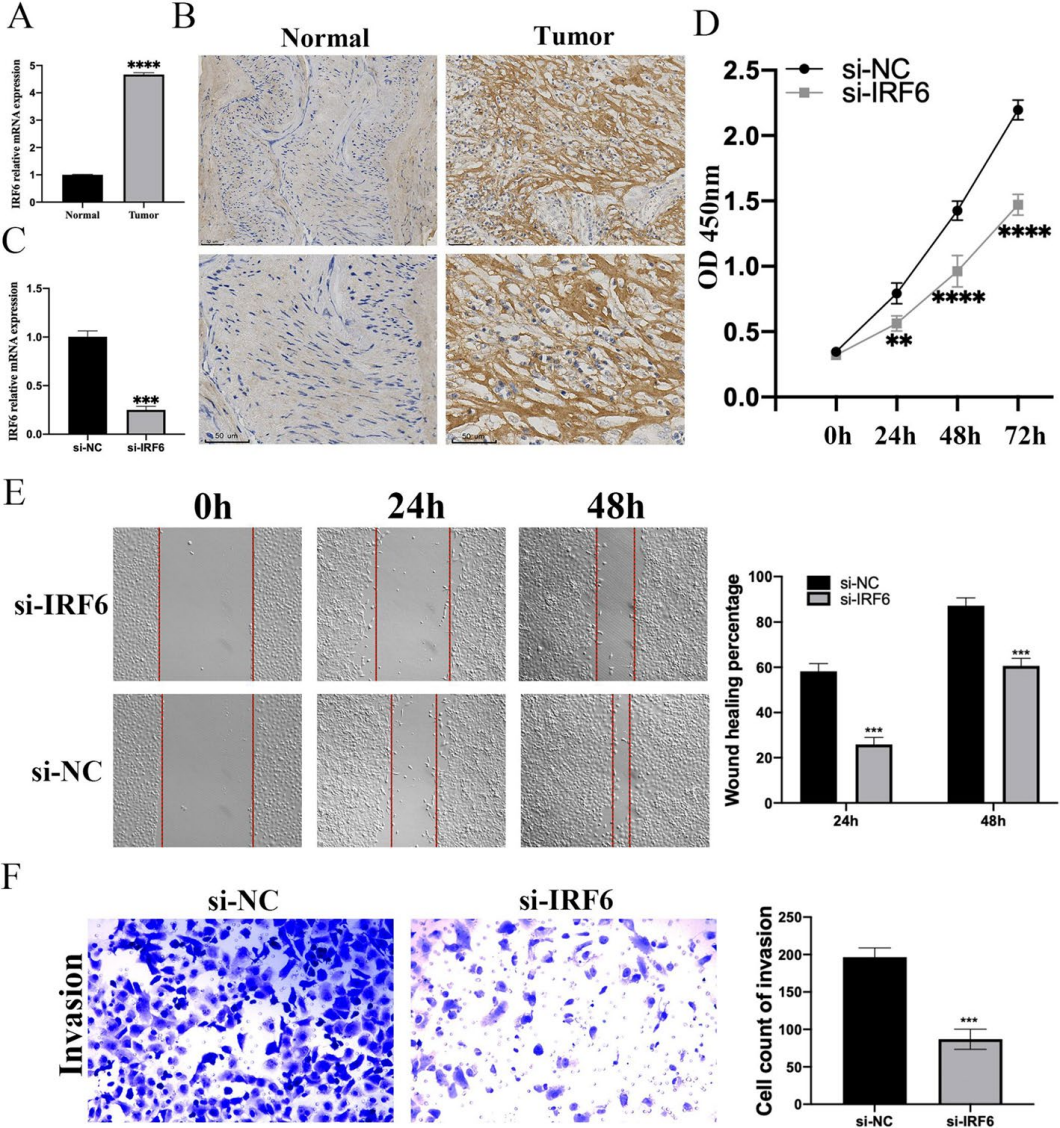
Experimental validation of IRF6. **A** The mRNA expression of IRF6 in OC tissues and adjacent normal tissues was measured by qRT-PCR (**B**) Immunohistochemistry of IRF6 based on the OC tissues and adjacent normal tissues. **C** The mRNA expression of IRF6 in SKOV3 cells was detected via qRT-PCR after transfection with siRNA targeting IRF6 for 48 h. **D** CCK8 experiment results of SKOV3 cells after cell transfection. **E** The scratch assay and statistical analysis of SKOV3 cells for the group original and si-IRF6. **F** The effect of IRF6 on invasion of SKOV3 cells was investigated by transwell invasion assay and statistical analysis. Data are mean ± SD from three independent experiments for D. The scales bar to indicate 50 μm for B. (**means *P* < 0.01; *** means *P* < 0.001, **** means *P* < 0.0001, *N* = 3, Student’s t-test, Error bars are ± SEM)

To validate our bioinformatics analysis results, we performed RT-qPCR analysis and immunohistochemical staining of IRF6, and the results revealed that the RNA and protein levels of IRF6 in tumors were higher than those in normal samples (Figs. 8A-B). To further verify the importance of IRF6 in OC, we constructed a human OC cell line SKOV3 that knocks down IRF6 to explore the effect of IRF6 on cell phenotypes of OC. We knocked down IRF6 by siRNAs (Fig. 8C). Data from the CCK8 detection assay suggested that IRF6 knockdown inhibited the proliferation of SKOV3 cells (Fig. 8D). The results of scratch wound assay demonstrated that knocking down IRF6 decreased the migration ability of SKOV3 cells (Fig. 8E). Similarly, the Transwell invasion assay significantly decreased invading cells after knocking down IRF6 (Fig. 8F). This finding indicates that IRF6 can regulate the migration and invasion of SKOV3 cell migration.

### Drug sensitivity

**Fig. 9:**
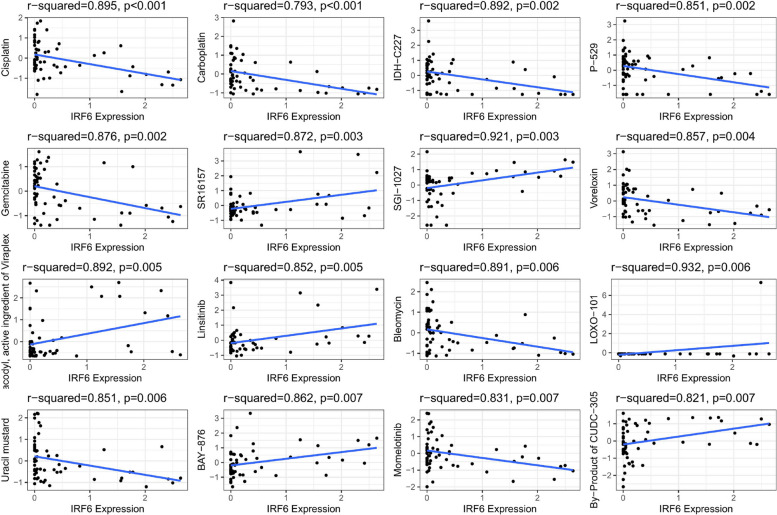
Drug sensitivity analysis. The expression of IRF6 was associated with the sensitivity of Cisplatin,Carboplatin,IDH-C227,P-529,Gemcitabine, SR16157, SGI-1027,Voreloxin, Bisacodyl acvtive i,Linsitinib, Bleomycin, LOXO-101,Uracil mustard, BAY-876,Momelotinib,By-product of CUDC-305.Downloaded from CellMiner were the molecular and pharmacological characteristics of NCI-60 cancer cells

Z-score-measured drug sensitivity was evaluated along with the mRNA expression level of IRF6. The most significantly correlated drugs are illustrated in Fig. 9, indicating that the increased expression level of IRF6 is related to the reduced sensitivity of cells to various DNA inhibitors, such as cisplatin, carboplatin, and gemcitabine, and increased sensitivity to various drugs, such as SGI-1027, Linsitinib, and BAY-876.

## Discussion

As one of the three common gynecological malignancies, OC has a high incidence rate and mortality [13]. Many factors affect the pathogenesis and prognosis of OC patients [14]. However, because of the lack of strong biomarkers, most OC patients are diagnosed at a later stage. The prognosis for patients with advanced OC is poor [15]. Although there has been improvement after receiving clinical treatment, the 5-year survival rate of patients with advanced OC is extremely low because of their high recurrence and cancer cell metastasis rate [7, 16]. Furthermore, the current treatment targets only apply to some OC patients, and the reasons for this situation remain unclear [17]. Therefore, screening for novel biomarkers of OC is crucial.

Recently, establishing various public databases and developing bioinformatics have promoted research on cancer pathogenesis. The STMN2, ESM1, and COL6A3 genes have been identified as novel biomarkers for OC patients [10, 18, 19]. Our study combined the differential gene analysis results of Limma and WGCNA, and through joint analysis of multiple datasets such as GSE66957, GSE14407 [20], GSE18520 [21], and TCGA + GTEx, we found that three genes (CLDN3, IRF6, and PRSS8) were closely related to the occurrence and development of OC. Survival analysis indicated that CLDN3 and IRF6 were associated with OS and PFS in OC patients, indicating that these genes have prognostic value. We used multiple algorithms to quantify the infiltration of immune cells in OC patients. In addition, ceRNA networks for CLDN3, IRF6, and PSRR8. CLDN3 and PSRR8 have been proven to have carcinogenic effects in OC, whereas IRF6 has not been reported in cancer. Therefore, we also validated the role of IRF6 in OC.

The Claudin family is a recently discovered type of tight junction protein, and changes in its expression and distribution directly affect the structure and function of tight junctions [22]. Among these, abnormal expression of Claudin-3 (CLDN3) is closely related to the occurrence and development of tumors [23]. CLDN3 is an essential cytoskeletal protein in tight junctions, and it is currently widely believed that the loss of intercellular adhesion can lead to the destruction of tight junctions, which is related to the infiltration and metastasis of tumor cells [24]. The primary function of CLDN3 is to maintain the physical barrier function between cells [25, 26]. The exact relationship between abnormal CLDN3 expression and tumors is still unclear. In colorectal cancer cells, the expression level of CLDN3 is abnormally increased, and its overexpression is associated with the deterioration of colorectal cancer [27]. In OC, CLDN3 overexpression may promote the invasion and movement of cancer cells [28]. Therefore, the specific role of CLDN3 may depend on the high specificity of the tissue and the precise molecular signaling pathway within the cell. Taken together, CLDN3 is one of the most highly upregulated OC pathogenic genes, which is consistent with our results [29].

In the present study, we used the KM plot to test the prognostic value of PRSS8; however, we did not find any association between PRSS8 and OS, PFS, and PPS in OC patients. However, according to existing reports, PRSS8 is an early diagnostic marker for OC, which may be due to inconsistent clinical information data [30]. Furthermore, the expression level of PRSS8 significantly increased in OC patients and cell lines, which is consistent with the results of our bioinformatics analysis [30]. Moreover, Xing Peng et al. found that knocking down PRSS8 inhibited the proliferation, migration, and EMT of OC cells, indicating that PRSS8 is a carcinogen of OC cells [31]. However, there is still some controversy over the role of PRSS8. For example, PRSS8 is a tumor suppressor in colorectal and liver cancer cells [32]. Therefore, we believe that PRSS8 may play different regulatory roles in other tumors.

IRF6 was the biggest gain of this study. As a member of the interferon regulatory family of DNA transcription factors, IRF6 encodes a highly conserved winged-helix DNA-binding protein [33]. IRF6 is primarily associated with human craniofacial anomalies, but its association with cancer has not been reported [34]. Although studies have reported a potential link between tooth agenesis and cancer, no such link has been established [35]. In the present study, we found that IRF6 RNA and protein levels were highly expressed in OC and were related to the OS and PFS of OC patients. Given the lack of research on IRF6 in OC, we conducted a preliminary exploration of its function in OC. The CCk8 assay, transwell migration and invasion experiments depicted that IRF6 knockdown inhibited the proliferation, migration and invasion abilities of the OC cell line SKOV3. This finding indicates that IRF6 may be a cancer-promoting factor in OC and become tumor markers in the future to guide OC patient prognosis recommendations.

However, our study has some shortcomings. First, we only used a small sample for IHC staining, which was insufficient for identifying IRF6 as a diagnostic and therapeutic biomarker for ovarian cancer patients. Second, the used dataset was not large enough and should include as many datasets as possible for analysis to determine the accuracy of IRF6 as a biomarker for ovarian cancer patients. Although we constructed a ceRNA network, it has not been studied. Finally, we only conducted a preliminary exploration of the role of IRF6 in ovarian cancer. However, soon, we will use more clinical samples and further experiments to verify our results.

## Conclusion

In summary, our research has identified a new target, IRF6, which may be related to the development of OC and may serve as an effective diagnostic marker for OC. In addition, we established a ceRNA network for IRF6 and predicted its association with sensitivity to various chemotherapeutic drugs. These findings could be applied in future clinical studies.

### Supplementary Information

**Supplementary Material 1.**

## Data Availability

No datasets were generated or analysed during the current study.
