# Supplementary material for: Identification and validation of IRF6 related to ovarian cancer and biological function and prognostic value
Source: J Ovarian Res. 2024 Mar 16;17:64. doi: 10.1186/s13048-024-01386-4 (PMC10943877; doi:10.1186/s13048-024-01386-4)
Supplement: Supplementary file 1 — Supplementary Material 1. [file 13048_2024_1386_MOESM1_ESM.zip › supplementary/Supplementary File 9.docx]

**Supplementary Table 1** De-identified metadata from 3 ovarian cancer patients and 3 cervical cancer patients

| Patient ID | Age | Race | Diagnosis | Histologic type | Surgical therapy |
| --- | --- | --- | --- | --- | --- |
| PT-1 | 48 | Asian | ovarian cancer | high grade serous carcinoma | cytoreductive surgery for ovarian cancer |
| PT-2 | 50 | Asian | ovarian cancer | high grade serous carcinoma cytoreductive surgery for ovarian cancer | |
| PT-3 | 46 | Asian | ovarian cancer | high grade serous carcinoma cytoreductive surgery for ovarian cancer | |
| PT-4 | 47 | Asian | cervical cancer | normal ovarian tissues | laparoscopic uterine bilateral adnexectomy |
| PT-5 | 45 | Asian | cervical cancer | normal ovarian tissues | laparoscopic uterine bilateral adnexectomy |
| PT-6 | 42 | Asian | cervical cancer | normal ovarian tissues | laparoscopic uterine bilateral adnexectomy |
